# Persistent infections, immune-senescence and Alzheimer's disease

**DOI:** 10.18632/oncoscience.309

**Published:** 2016-06-30

**Authors:** Federico Licastro, Elisa Porcellini

**Affiliations:** ^1^ Department of Experimental, Diagnostic and Specialty Medicine, School of Medicine, University of Bologna, Bologna 40126, Italy

**Keywords:** Alzheimer's disease, herpes virus latency and infection, peripheral inflammation, neuro-inflammation and cognitive decline

## Abstract

Alzheimer's disease (AD) is a progressive neurodegenerative disorder and the most common cause of dementia. Classical hallmarks of AD such as amyloid deposition and neurofibrillary tangles do not completely explain AD pathogenesis. Recent investigations proposed Aβ peptide as an anti-microbial factor. Our previous works suggested that the concomitant presence of single nucleotide polymorphisms (SNPs) from AD genetic studies might impair antiviral defenses and increase the individual susceptibility to herpes virus infection. Viruses of herpes family by inducing frequent cycles of reactivation and latency constantly challenge the immune response and drive the accumulation of memory T cells. However, the immune system is not able to completely eradicate these viruses. The continuous antigen stimulation activates chronic inflammatory responses that may progressively induce neurodegenerative mechanisms in genetically susceptible elderly. The aim of this paper is to suggest new perspectives in clinical pathogenesis of AD with potential prevention and new medical treatment of the age associated cognitive decline.

## INTRODUCTION

Alzheimer's disease (AD) is a progressive neurodegenerative disorder and represents the most common cause of dementia. Neuropathological alterations, such as amyloid deposition and neurofibrillary tangles (NFTs) are autopsy hallmarks found in the brain of AD patients and these alterations have been suggested to be causative of the disease [1, 2]. However, amyloid plaques and NFTs are also present in the brain of elderly who died without the clinical presentation of AD [3]; therefore, the notion that amyloid deposition and other proteinaceous alterations might be linked to the etiology of AD remains uncertain.

Because of the urgency for effective preventive and therapeutic measures, extensive research has focused on pathogenetic mechanisms of AD; however, up to now, no therapy has been found. Therefore, it is now relevant to focus upon new components of the disease pathogenesis in order to discover new therapeutic strategies for patients and prevention opportunities for the elderly without manifest cognitive alterations, but with increased risk of developing dementia.

The physiological function of the amyloid precursor protein (APP) and the biological role of its proteolytic derivatives are still unclear [4]. However, A-beta peptide has been suggested to play a role as anti- microbial defensive factor [5–8] and recent investigations confirmed that the A-beta peptides showed a relevant anti- viral activity *in vitro* [9, 10] and a role in innate immune responses, A-beta being involved in microglia activation [11]. These observations indicated that A-beta peptides may play a defensive role against microorganisms.

In previous publications [12–14] we discussed genetic data from four genome wide association (GWA) studies on AD [15–18]. From these investigations a set of single-nucleotide polymorphisms (SNPs) associated with AD emerged and we suggest that the concomitant presence of several of these SNPs in a single person might result in a genetic signature predisposing to AD, via complex and diverse mechanisms, each contributing to an increase of individual susceptibility to herpes virus infection [12].

Among environmental factors potentially associated with the age related cognitive decline, persistent virus infection along with the progressive decline of immune competence with advancing age may play a pivotal role in AD.

### Herpes simplex virus type 1 (HSV-1) and AD

HSV-1 is a neurotropic virus that infects a large part of human population. A viral etiology, especially involving herpes virus in AD, has been already proposed and most investigations have shown an association of HSV-1 with AD [19–21]. It is of interest that Letenneur et al., showed an association of anti HSV-1 IgM levels and incidental AD in a 512 elderly cohort [22].

Moreover, recent reports showed a significant association of HSV-1 infection with AD risk [23]. In fact, a reactivation of HSV-1 infection assessed by increased serum levels of specific anti-HSV-1 antibodies was found associated with an increased AD risk in a longitudinal study on 3,432 Swedish elderly [23]. Another study from Italy reported that elevated serum HSV-1 antibody titers correlated with cortical grey matter volume, as assessed by MRI [24].

Some studies have suggested that in people carrying the APOE-ε4 allele and, therefore, predisposed to develop AD, HSV-1 infection markedly increases the risk of AD [25–28]. This hypothesis was also supported by transgenic mice experiments [29].

### Cytomegalovirus (CMV) and AD

CMV is ubiquitously distributed in human population and the most frequent cause of brain infection in immune compromised patients or in infants with congenital virus transmission [30, 31]. In the majority of human population, postnatal acute peripheral CMV infection is usually asymptomatic, however, the virus, once established, remains latent in blood monocytes [32, 33]. Several lines of evidences indicated that CMV may be a risk factor for AD. For instance, an increased rate of cognitive decline over a four year period in subjects with elevated CMV antibody levels has been reported [34]

Several other studies have reported the association of CMV and cognitive impairment; however, results have been conflicting [35–38].

A previous work upon brain frontal and temporal cortex samples found that both AD patients and elderly healthy subjects were positive for CMV with no statistically significant difference between the two groups [39]. On the other hand, brain positivity for CMV was found in a greater proportion of patients with vascular dementia than normal elderly and these findings suggested a virus role in the disease [40]. CMV was also present in CSF of subjects with encephalitis or meningitis or other neurological condition [41].

A recent investigation reported increased CMV antibody levels in the elderly who developed clinical AD during a five years follow up [14]. Furthermore, findings from a longitudinal follow up of 849 participants in the USA showed that CMV infection doubled the risk of developing AD [42], even if some criticisms to the above data have been presented [43].

### Epstein-Barr Virus (EBV) and AD

EBV infects more than 95% of human beings within the first years of life. The virus is the agent of acute infectious mononucleosis in a minority of immune competent subjects, while the majority develops a lifelong asymptomatic infection, with the virus remaining latent in B-lymphocytes [44–46].

Data describing an association between EBV and AD are very limited; only two papers from 1992 described a possible correlation between EBV and AD, however, with discordant results [47–48].

Recently, our findings showed a positive association of peripheral blood positivity for EBV genome and AD and elevated levels of EBV specific antibodies positively associated with an increased risk of developing AD [14].

### Human herpes virus (HHV)-6 and AD

HHV-6 is a neurotropic virus, present in two different variants [49] with a very high sero-prevalence involving almost 100% of population by the age three [50]. HHV-6 establishes latency in the brain and may reactivate under conditions of immunosuppression [49].

HHV-6 has been associated with multiple neurological diseases including seizures, encephalitis, mesial temporal lobe epilepsy and multiple sclerosis [51].

HHV-6 has been found in a higher proportion of AD than age-matched control (CTR) brains [39]. Recently, Agostini et al. showed no difference in serum HHV-6 IgG antibody titers and avidity index between AD patients, Mild Cognitive Impairment subjects (MCI) and CTRs [52]. Another study showed similar results where a higher value of HHV-6 levels in CTR brains was found [53].

Our findings showed an elevated positivity in brains and the peripheral blood for HHV-6 genome in AD and an increased sero-positivity associated with increased risk of developing AD [14].

## THE AGE-DEPENDENT RESHAPING OF IMMUNE RESPONSES INDUCED BY PERSISTENT VIRUS INFECTIONS

The sero-positivity to CMV, EBV or HHV-6 is very high worldwide and viruses of the herpes family are largely and commonly present in the elderly [54–56].

The aging of the immune system is a continuous and dynamic process and it may be secondary to mechanisms activated by the response to the pathogen individual internal milieu.

Innate immune response is partially affected by human aging. In fact, a decrease in the main functions of innate immunity cells, as a consequence of changes in the expression of a variety of innate immune cell receptors and altered signal transduction pathways have been reported. These defects may result in a reduced capacity to respond against bacterial and viral pathogens [57].

Adaptive immune responses also progressively decline with age [58]. Recent investigations focused on immune senescence suggested that the progressive decline of immune defense efficiency might be an adaptation mechanism to the microorganism exposure experienced by the aging organism over the life time [38, 59–62].

Human Immune deficiency (HIV) virus along with human hepatitis B virus (HBV), EBV, varicella zoster virus (VHZ) and HSV-1 have a severe impact on immune system and contribute to reshape the immune phenotype in the old person by inducing a persistent antigenic stimulation [63].

For instance, viruses of the herpes family, by undergoing frequent cycles of reactivation and latency drive the accumulation of memory T cells; however, the immune system is not able to completely eradicate the viruses. In fact, the continuous antigen stimulation induced by persistent infections activates a peripheral chronic inflammatory response that progressively induces the loss of naïve and inducible CD4 and CD8 positive T cells, along with the accumulation of memory T cell populations; however, most of these cells are considered functionally exhausted [59, 64–67].

Therefore, chronic sub-clinical infections represent important environmental factors able to induce a re-shaping of the immune system by antigen load during aging.

## PERIPHERAL CHRONIC SUBCLINICAL INFECTION AND AD

A recent overview on immune responses and AD concluded that, in spite of conflicting data, blood levels of some cytokines showed a steady increase during progression from MCI to AD [68]. These findings were confirmed by another study [69].

Increased serum levels of inflammatory factors have been reported also in MCI from Chinese patients [70]. An association between late life depression, MCI and AD is well documented and some findings suggested that peripheral inflammation might be the missing link in these different conditions [71–73].

Investigation from the Rush Alzheimer's Disease Center Religious Order showed that CMV serum IgG levels correlated with NFT in the autopsy brains [32]. It is of interest that the percentages of senescent CD4 and CD8 positive T cells were higher in CMV sero-positive than in sero-negative subjects and marginally associated with AD diagnosis.

Moreover, Lurain and co-workers reported that the infection of human fibroblasts by CMV induced the expression of amyloid beta peptides [32]. Therefore, a more stringent link between peripheral and central inflammatory responses in AD due to infective agents is now emerging [74–75].

This link may consist of chronic infections by microorganisms, such as viruses, that are able to constantly challenge and impair immune responses.

## BRAIN IMMUNE RESPONSES AND AD

Microglia activation in pre-clinical and clinical AD by neuro-imaging techniques has been reported [76-79]. Brain microglia from AD patients is activated and release several cytokines which drive neuro-inflammation [80, 81]. A recent paper reviewed this topic suggesting that brain infiltrating T cells may stimulate microglia activation by releasing IFN-gamma and, therefore, influence neuro-degenerative processes associated with AD [82]. These findings were confirmed by Browne et al. who suggested that release of IFN-γ from infiltrating Th1 cells significantly accelerated the accumulation of markers of the disease in an animal model of AD [83]. A defective resolution of inflammatory state has been recently found in the brain of patients with AD and such an impairment correlated with cognitive performances [84]. Moreover, elevated levels of CNS inflammation and CSF inflammatory markers have also been reported in preclinical stages of AD [85].

Recent findings reinforced the notion that brain inflammation, as assessed by CSF markers, increased in normal aging and was associated with markers of neurodegeneration in the preclinical stages of AD [86].

Activated brain microglia increased during aging and AD brain microglia might be primed by infectious agents challenging the CNS and/or by temporary permeabilization of selected districts of blood-brain barrier (BBB) induced by peripheral subclinical inflammatory responses.

Virus infections are not the unique challenge for the aging immune system. In fact persistent bacterial infections may also play a role in inducing chronic inflammation in the elderly. It is of interest that chronic infections, by different bacterial agents have been recently implicated in AD pathogenesis. A recent review, by Harris et al., confirmed that infection agents such as CMV, HSV-1, HHV-6, *Helicobacter pylori*, *Chlamydophila pneumonia* and several periodontal pathogens induced the production of peripheral pro-inflammatory cytokines that, by crossing the BBB may, promote neurodegeneration [87]. In addition, other bacterial agents, such as spirochetes, have also been proposed to be associated with AD [88]. Since spirochetes frequently co-infect with other microorganisms, Miklossy et al. suggested that chronic infection by spirochetes, and co-infection with other bacteria and viruses should be considered in AD [88].

Oral infections have been recently reviewed as potential causes of BBB disruption and brain inflammation. These pathogens may also infect the brain via trigeminal and/or olfactory nerves [87, 89].

It is of interest that a recent investigation showed a significant improvement in some cognitive functions in patients with early AD treated for twenty eight weeks with interferon beta-1 [90]. This compound is a well known anti-viral agent and the above findings support the notion suggesting a potential role of persistent virus infections in the disease.

## CONCLUSIONS

**Figure 1:**
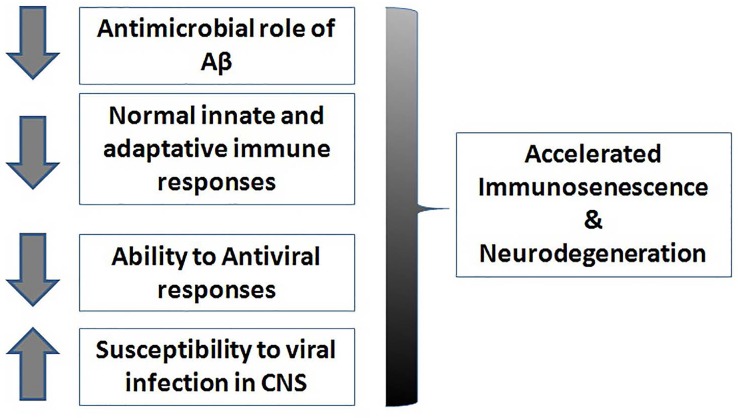
Risk factors related to defective immune responses leading to neurodegeneration. A decrease of the antimicrobial role of A-beta, a decline of normal immune responses and a decreased ability of antiviral responses associated to an increased susceptibility to infections can lead to an accelerated immunosenescence and to neurodegeneration. CNS (central nervous system); Aβ (Α−beta peptide).

AD is a multi-factorial disease in which, several pathogenetic, clinical, environmental and stochastic factors are involved (Fig. 1). It is on record that defective immune responses (both innate and adaptive) resulting in a chronic inflammatory “status” of the brain might lead to neurodegenerative disease such as AD. With this short overview we focus on the pivotal role of infective agents in AD. In fact, several pathogens are able to induce a reshaping of adaptive immune responses and to impair the regulation of both peripheral and central immune defensive mechanisms. In particular, defective immune defenses against some pathogens, both viruses and bacteria, may play a role in triggering chronic inflammatory responses and directly or indirectly activate neuro-inflammation. [91] The activation of persistent peripheral inflammation may also be detrimental in the brain in genetically susceptible individuals. According to this view, APP and its peptides derivatives, normally defined as promoter of neurodegeneration, appear to play an important role in brain defenses against microorganisms since these molecules show antimicrobial activity both *in vivo* and *in vitro*. Here we suggest that in subjects developing clinical AD, immune protective mechanisms appear to be defective. Therefore, persistent subclinical infections activate and amplify chronic neuroinflammation and neurodegenerative mechanisms leading to progressive neuronal loss and cognitive impairment. Successful treatment of chronic infections is a challenge, but might significantly improve the quality of life in the elderly and prevent or retard the age associated cognitive decline leading to dementia.

## References

[1] Terry RD (1994). Neuropathological changes in Alzheimer disease. Prog Brain Res.

[2] Trojanowski JQ, Clark CM, Schmidt ML, Arnold SE, Lee VM (1997). Strategies for improving the postmortem neuropathological diagnosis of Alzheimer's disease. Neurobiol Aging.

[3] Elman JA, Oh H, Madison CM, Baker SL, Vogel JW, Marks SM, Crowley S, O'Neil JP, Jagust WJ (2014). Neural compensation in older people with brain amyloid-β deposition. Nat Neurosci.

[4] Nalivaeva NN, Turner AJ (2013). The amyloid precursor protein: a biochemical enigma in brain development, function and disease. FEBS Lett.

[5] Soscia SJ, Kirby JE, Washicosky KJ, Tucker SM, Ingelsson M, Hyman B, Burton MA, Goldstein LE, Duong S, Tanzi RE, Moir RD (2010). The Alzheimer's disease-associated amyloid beta-protein is an antimicrobial peptide. PLoS One.

[6] Chakrabarti A (2007). Epidemiology of central nervous system mycoses. Neurology India.

[7] Cherny RA, Legg JT, McLean CA, Fairlie DP, Huang X, Atwood CS, Beyreuther K, Tanzi RE, Masters CL, Bush AI (1999). Aqueous dissolution of Alzheimer's disease Aβ amyloid deposits by biometal depletion. J Biol Chem.

[8] McCafferty DG, Cudic P, Yu MK, Behenna DC, Kruger R (1999). Synergy and duality in peptide antibiotic mechanisms. Curr Opin Chem Biol.

[9] White MR, Kandel R, Tripathi S, Condon D, Qi L, Taubenberger J, Hartshorn KL (2014). Alzheimer's associated β-amyloid protein inhibits influenza A virus and modulates viral interactions with phagocytes. PLoS One.

[10] Bourgade K, Garneau H, Giroux G, Le Page AY, Bocti C, Dupuis G, Frost EH, Fülöp T (2015). β-Amyloid peptides display protective activity against the human Alzheimer's disease-associated herpes simplex virus-1. Biogerontology.

[11] Carrano A, Das P (2015). Altered Innate Immune and Glial Cell Responses to Inflammatory Stimuli in Amyloid Precursor Protein Knockout Mice. PLoS One.

[12] Licastro F, Carbone I, Ianni M, Porcellini E (2011). Gene signature in Alzheimer's disease and environmental factors: the virus chronicle. J Alzheimers Dis.

[13] Porcellini E, Carbone I, Ianni M, Licastro F (2010). Alzheimer's disease gene signature says: beware of brain viral infections. Immun Ageing.

[14] Carbone I, Lazzarotto T, Ianni M, Porcellini E, Forti P, Masliah E, Gabrielli L, Licastro F (2014). Herpes virus in Alzheimer's disease: relation to progression of the disease. Neurobiol Aging.

[15] Lambert JC, Heath S, Even G, Campion D, Sleegers K, Hiltunen M, Combarros O, Zelenika D, Bullido MJ, Tavernier B, Letenneur L, Bettens K, Berr C (2009). Genome- wide association study identifies variants at CLU and CR1 associated with Alzheimer's disease. Nat Genet.

[16] Hollingworth P, Harold D, Sims R, Gerrish A, Lambert JC, Carrasquillo MM, Abraham R, Hamshere ML, Pahwa JS, Moskvina V, Dowzell K, Jones N, Stretton A (2011). Common variants at ABCA7, MS4A6A/MS4A4E, EPHA1, CD33 and CD2AP are associated with Alzheimer's disease. Nat Genet.

[17] Naj AC, Jun G, Beecham GW, Wang LS, Vardarajan BN, Buros J, Gallins PJ, Buxbaum JD, Jarvik GP, Crane PK, Larson EB, Bird TD, Boeve BF (2011). Common variants at MS4A4/MS4A6E, CD2AP, CD33 and EPHA1 are associated with late-onset Alzheimer's disease. Nat Genet.

[18] Harold D, Abraham R, Hollingworth P, Sims R, Gerrish A, Hamshere ML, Pahwa JS, Moskvina V, Dowzell K, Williams A, Jones N, Thomas C, Stretton A (2009). Genome- wide association study identifies variants at CLU and PICALM associated with Alzheimer's disease. Nat Genet.

[19] Jamieson GA, Maitland NJ, Wilcock GK, Craske J, Itzhaki RF (1991). Latent herpes simplex virus type 1 in normal and Alzheimer's disease brains. J Med Virol.

[20] Wozniak MA, Mee AP, Itzhaki RF (2009). Herpes simplex virus type 1 DNA is located within Alzheimer's disease amyloid plaques. J Pathol.

[21] Wozniak MA, Frost AL, Itzhaki RF (2009). Alzheimer's disease-specific tau phosphorylation is induced by herpes simplex virus type 1. J Alzheimers Dis.

[22] Letenneur L, Pérès K, Fleury H, Garrigue I, Barberger-Gateau P, Helmer C, Orgogozo JM, Gauthier S, Dartigues JF (2008). Seropositivity to herpes simplex virus antibodies and risk of Alzheimer's disease: A populationbased cohort study. PLoS One.

[23] Lövheim H, Gilthorpe J, Johansson A, Eriksson S, Hallmans G, Elgh F (2015). Herpes simplex infection and the risk of Alzheimer's disease-A nested case-control study. Alzheimers Dement.

[24] Mancuso R, Baglio F, Cabinio M, Calabrese E, Hernis A, Nemni R, Clerici M (2014). Titers of herpes simplex virus type 1 antibodies positively correlate with grey matter volumes in Alzheimer's disease. J Alzheimers Dis.

[25] Itzhaki RF, Lin WR, Shang D, Wilcock GK, Faragher B, Jamieson GA (1997). Herpes simplex virus type 1 in brain and risk of Alzheimer's disease. Lancet.

[26] Itzhaki RF, Lin WR (1998). Herpes simplex virus type I in brain and the type 4 allele of the apolipoprotein E gene are a combined risk factor for Alzheimer's disease. Biochem Soc Trans.

[27] Beffert U, Bertrand P, Champagne D, Gauthier S, Poirier J (1998). HSV-1 in brain and risk of Alzheimer's disease. Lancet.

[28] Strandberg TE, Pitkala K, Eerola J, Tilvis R, Tienari PJ (2005). Interaction of herpesviridae, APOE gene, and education in cognitive impairment. Neurobiol Aging.

[29] Burgos JS, Ramirez C, Sastre I, Valdivieso F (2007). Apolipoprotein E genotype influences vertical transmission of herpes simplex virus type 1 in a gender specific manner. Aging Cell.

[30] Tsutsui Y, Kosugi I, Kawasaki H, Arai Y, Han GP, Li L, Kaneta M (2008). Roles of neural stem progenitor cells in cytomegalovirus infection of the brain in mouse models. Pathol Int.

[31] Pawelec G, Derhovanessian E, Larbi A, Strindhall J, Wikby A (2009). Cytomegalovirus and human immunosenescence. Rev Med Virol.

[32] Lurain NS, Hanson BA, Martinson J, Leurgans SE, Landay AL, Bennett DA, Schneider JA (2013). Virological and immunological characteristics of human cytomegalovirus infection associated with Alzheimer disease. J Infect Dis.

[33] Taylor-Wiedeman J, Sissons JG, Borysiewicz LK, Sinclair JH (1991). Monocytes are a major site of persistence of human cytomegalovirus in peripheral blood mononuclear cells. J Gen Virol.

[34] Aiello AE, Haan M, Blythe L, Moore K, Gonzalez JM, Jagust W (2006). The influence of latent viral infection on rate of cognitive decline over 4 years. J Am Geriatr Soc.

[35] Tarter KD, Simanek AM, Dowd JB, Aiello AE (2014). Persistent viral pathogens and cognitive impairment across the lifecourse in the third National health and nutrition examination survey. J Infect Dis.

[36] Mathei C, Vaes B, Wallemacq P, Degryse J (2011). Associations between cytomegalovirus infection and functional impairment and frailty in the BELFRAIL Cohort. J Am Geriatr Soc.

[37] Gow AJ, Firth CM, Harrison R, Starr JM, Moss P, Deary IJ (2013). Cytomegalovirus infection and cognitive abilities in old age. Neurobiol Aging.

[38] Stowe RP, Peek MK, Cutchin MP, Goodwin JS (2012). Reactivation of herpes simplex virus type 1 is associated with cytomegalovirus and age. J Med Virol.

[39] Lin WR, Wozniak MA, Cooper RJ, Wilcock GK, Itzhaki RF (2002). Herpesviruses in brain and Alzheimer's disease. J Pathol.

[40] Lin WR, Wozniak MA, Wilcock GK, Itzhaki RF (2002). Cytomegalovirus is present in a very high proportion of brains from vascular dementia patients. Neurobiol Dis.

[41] Studahl M, Hagberg L, Rekabdar E, Bergström T (2000). Herpesvirus DNA detection in cerebral spinal fluid: differences in clinical presentation between alpha-, beta-, and gamma-herpesviruses. Scand J Infect Dis.

[42] Barnes LL, Capuano AW, Aiello AE, Turner AD, Yolken RH, Torrey EF, Bennett DA (2015). Cytomegalovirus infection and risk of Alzheimer disease in older black and white individuals. J Infect Dis.

[43] Itzhaki RF, Klapper P (2014). Cytomegalovirus: an improbable cause of Alzheimer disease. J Infect Dis.

[44] Licastro F, Carbone I, Raschi E, Porcellini E (2014). The 21st century epidemic: Infections as inductors of neuro- degeneration associated with Alzheimer's disease. Immun Ageing.

[45] Landais E, Saulquin X, Houssaint E (2005). The human T cell immune response to Epstein-Barr virus. Int J Dev Biol.

[46] Kurth J, Spieker T, Wustrow J, Strickler GJ, Hansmann LM, Rajewsky K, Küppers R (2000). EBV-infected B cells in infectious mononucleosis: viral strategies for spreading in the B cell compartment and establishing latency. Immunity.

[47] Kittur SD, Hoh JH, Kawas CH, Hayward GS, Endo H, Adler WH (1992). A molecular hybridization study for the presence of Herpes simplex, cytomegalovirus and Epstein-Barr virus in brain and blood of Alzheimer's disease patients. Arch Gerontol Geriatr.

[48] Ounanian A, Guilbert B, Seigneurin JM (1992). Characteristics of Epstein-Barr virus transformed B cell lines from patients with Alzheimer's disease and age-matched controls. Mech Ageing Dev.

[49] Yao K, Gagnon S, Akhyani N, Williams E, Fotheringham J, Frohman E, Stuve O, Monson N, Racke MK, Jacobson S (2008). Reactivation of human herpesvirus-6 in natalizumab treated multiple sclerosis patients. PLoS One.

[50] Stone RC, Micali GA, Schwartz RA (2014). Roseola infantum and its causal human herpesviruses. Int J Dermatol.

[51] Yao K, Crawford JR, Komaroff AL, Ablashi DV, Jacobson S (2010). Review part 2: Human herpesvirus-6 in central nervous system diseases. J Med Virol.

[52] Agostini S, Mancuso R, Baglio F, Cabinio M, Hernis A, Calabrese E, Nemni R, Clerici M (2015). Lack of Evidence for a Role of HHV-6 in the Pathogenesis of Alzheimer's Disease. J Alzheimers Dis.

[53] Hemling N, Röyttä M, Rinne J, Pöllänen P, Broberg E, Tapio V, Vahlberg T, Hukkanen V (2003). Herpesviruses in brains in Alzheimer's and Parkinson's diseases. Ann Neurol.

[54] Pawelec G (2014). Immunosenenescence: role of cytomegalovirus. Exp Gerontol.

[55] Schmader KE1, van der Horst CM, Klotman ME (1989). Epstein-Barr virus and the elderly host. Rev Infect Dis.

[56] Monastero R, Caruso C, Vasto S (2014). Alzheimer's disease and infections, where we stand and where we go. Immun Ageing.

[57] Solana R, Tarazona R, Gayoso I, Lesur O, Dupuis G, Fulop T (2012). Innate immunosenescence: effect of aging on cells and receptors of the innate immune system in humans. Semin Immunol.

[58] Solana R, Tarazona R, Aiello AE, Akbar AN, Appay V, Beswick M, Bosch JA, Campos C, Cantisán S, Cicin-Sain L, Derhovanessian E, Ferrando-Martínez S, Frasca D (2012). CMV and Immunosenescence: from basics to clinics. Immun Ageing.

[59] Fülöp T, Larbi A, Pawelec G (2013). Human T cell aging and the impact of persistent viral infections. Front Immunol.

[60] Stowe RP, Kozlova EV, Yetman DL, Walling DM, Goodwin JS, Glaser R (2007). Chronic herpesvirus reactivation occurs in aging. Exp Gerontol.

[61] Almanzar G, Schwaiger S, Jenewein B, Keller M, Herndler-Brandstetter D, Würzner R, Schönitzer D, Grubeck-Loebenstein B (2005). Long-term cytomegalovirus infection leads to significant changes in the composition of the CD8+ T-cell repertoire, which may be the basis for an imbalance in the cytokine production profile in elderly persons. J Virol.

[62] Westman G, Berglund D, Widén J, Ingelsson M, Korsgren O, Lannfelt L, Sehlin D, Lidehall AK, Eriksson BM (2014). Increased inflammatory response in cytomegalovirus seropositive patients with Alzheimer's disease. PLoS One.

[63] Buchholz VR, Neuenhahn M, Busch DH (2011). CD8+ T cell differentiation in the aging immune system: until the last clone standing. Curr Opin Immunol.

[64] Buchholz VR, Gräf P, Busch DH (2013). The smallest unit: effector and memory CD8(+) T cell differentiation on the single cell level. Front Immunol.

[65] Olsson J, Wikby A, Johansson B, Löfgren S, Nilsson BO, Ferguson FG (2000). Age-related change in peripheral blood T-lymphocyte subpopulations and cytomegalovirus infection in the very old: the Swedish longitudinal OCTO immune study. Mech Ageing Dev.

[66] Wikby A, Ferguson F, Forsey R, Thompson J, Strindhall J, Löfgren S, Nilsson BO, Ernerudh J, Pawelec G, Johansson B (2005). An immune risk phenotype, cognitive impairment, and survival in very late life: impact of allostatic load in Swedish octogenarian and nonagenarian humans. J Gerontol A Biol Sci Med Sci.

[67] Hadrup SR, Strindhall J, Køllgaard T, Seremet T, Johansson B, Pawelec G, Thor Straten P, Wikby A (2006). Longitudinal studies of clonally expanded CD8 T cells reveal a repertoire shrinkage predicting mortality and an increased number of dysfunctional cytomegalovirus-specific T cells in the very elderly. J Immunol.

[68] Brosseron F, Krauthausen M, Kummer M, Heneka MT (2014). Body fluid cytokine levels in mild cognitive impairment and Alzheimer's disease: a comparative overview. Mol Neurobiol.

[69] Dursun E, Gezen-Ak D, Hanağası H, Bilgiç B, Lohmann E, Ertan S, Atasoy İL, Alaylıoğlu M, Araz ÖS, Önal B, Gündüz A, Apaydın H, Kızıltan G (2015). The interleukin 1 alpha, interleukin 1 beta, interleukin 6 and alpha-2-macroglobulin serum levels in patients with early or late onset Alzheimer's disease, mild cognitive impairment or Parkinson's disease. J Neuroimmunol.

[70] Zhao SJ, Guo CN, Wang MQ, Chen WJ, Zhao YB (2012). Serum levels of inflammation factors and cognitive performance in amnestic mild cognitive impairment: a Chinese clinical study. Cytokine.

[71] Hermida AP, McDonald WM, Steenland K, Levey A (2012). The association between late-life depression, mild cognitive impairment and dementia: is inflammation the missing link?. Expert Rev Neurother.

[72] Johnson LA, Gamboa A, Vintimilla R, Cheatwood AJ, Grant A, Trivedi A, Edwards M, Hall JR, O'Bryant SE (2015). Comorbid Depression and Diabetes as a Risk for Mild Cognitive Impairment and Alzheimer's Disease in Elderly Mexican Americans. J Alzheimers Dis.

[73] Van der Mussele S, Fransen E, Struyfs H, Luyckx J, Mariën P, Saerens J, Somers N, Goeman J, De Deyn PP, Engelborghs S (2014). Depression in mild cognitive impairment is associated with progression to Alzheimer's disease: a longitudinal study. J Alzheimers Dis.

[74] Bu XL, Yao XQ, Jiao SS, Zeng F, Liu YH, Xiang Y, Liang CR, Wang QH, Wang X, Cao HY, Yi X, Deng B, Liu CH (2015). A study on the association between infectious burden and Alzheimer's disease. Eur J Neurol.

[75] Westman G, Berglund D, Widén J, Ingelsson M, Korsgren O, Lannfelt L, Sehlin D, Lidehall AK, Eriksson BM (2014). Increased inflammatory response in cytomegalovirus seropositive patients with Alzheimer's disease. PLoS One.

[76] Schuitemaker A, Kropholler MA, Boellaard R, van der Flier WM, Kloet RW, van der Doef TF, Knol DL, Windhorst AD, Luurtsema G, Barkhof F, Jonker C, Lammertsma AA, Scheltens P (2013). Microglial activation in Alzheimer's disease: an (R)-[11C]PK11195 positron emission tomography study. Neurobiol Aging.

[77] Zimmer ER, Leuzy A, Benedet AL, Breitner J, Gauthier S, Rosa-Neto P (2014). Tracking neuroinflammation in Alzheimer's disease: the role of positron emission tomography imaging. J Neuroinflammation.

[78] Lautner R, Mattsson N, Schöll M, Augutis K, Blennow K, Olsson B, Zetterberg H (2011). Biomarkers for microglial activation in Alzheimer's disease. Int J Alzheimers Dis.

[79] McGeer PL, Itagaki S, Tago H, McGeer EG (1987). Reactive microglia in patients with senile dementia of the Alzheimer type are positive for the histocompatibility glycoprotein HLA-DR. Neurosci Lett.

[80] McGeer PL, Rogers J (1992). Anti-inflammatory agents as a therapeutic approach to Alzheimer's disease. Neurology.

[81] Griffin WS, Stanley LC, Ling C, White L, MacLeod V, Perrot LJ, White CL, Araoz C (1989). Brain interleukin 1 and S-100 immunoreactivity are elevated in Down syndrome and Alzheimer disease. Proc Natl Acad Sci U S A.

[82] Lynch MA (2014). The impact of neuroimmune changes on development of amyloid pathology; relevance to Alzheimer's disease. Immunology.

[83] Browne TC, McQuillan K, McManus RM, O'Reilly JA, Mills KH, Lynch MA (2013). IFN-γ Production by amyloid β-specific Th1 cells promotes microglial activation and increases plaque burden in a mouse model of Alzheimer's disease. J Immunol.

[84] Wang X, Zhu M, Hjorth E, Cortés-Toro V, Eyjolfsdottir H, Graff C, Nennesmo I, Palmblad J, Eriksdotter M, Sambamurti K, Fitzgerald JM, Serhan CN, Granholm AC (2015). Resolution of inflammation is altered in Alzheimer's disease. Alzheimers Dement.

[85] Monson NL, Ireland SJ, Ligocki AJ, Chen D, Rounds WH, Li M, Huebinger RM, Munro Cullum C, Greenberg BM, Stowe AM, Zhang R (2014). Elevated CNS inflammation in patients with preclinical Alzheimer's disease. J Cereb Blood Flow Metab.

[86] Alcolea D, Martínez-Lage P, Sánchez-Juan P, Olazarán J, Antúnez C, Izagirre A, Ecay-Torres M, Estanga A, Clerigué M, Guisasola MC, Sánchez Ruiz D, Marín Muñoz J, Calero M (2015). Amyloid precursor protein metabolism and inflammation markers in preclinical Alzheimer disease. Neurology.

[87] Harris SA, Harris EA (2015). Herpes Simplex Virus Type 1 and Other Pathogens are Key Causative Factors in Sporadic Alzheimer's Disease. J Alzheimers Dis.

[88] Miklossy J (2015). Historic evidence to support a causal relationship between spirochetal infections and Alzheimer's disease. Front Aging Neurosci.

[89] Shoemark DK, Allen SJ (2015). The microbiome and disease: reviewing the links between the oral microbiome, aging, and Alzheimer's disease. J Alzheimers Dis.

[90] Grimaldi LM, Zappalà G, Iemolo F, Castellano AE, Ruggieri S, Bruno G, Paolillo A (2014). A pilot study on the use of interferon beta-1a in early Alzheimer's disease subjects. J. Neuroinflammation.

[91] Miklossy J (2011). Emerging roles of pathogens in Alzheimer disease. Expert Rev Mol Med.

